# Efficient Hybrid Environment Expression for Look-and-Step Behavior of Bipedal Walking

**DOI:** 10.34133/cbsystems.0244

**Published:** 2025-04-23

**Authors:** Chao Li, Qingqing Li, Junhang Lai, Xuechao Chen, Zhangguo Yu, Zhihong Jiang

**Affiliations:** School of Mechatronic Engineering, Beijing Institute of Technology, Beijing 100081, P. R. China.

## Abstract

The look-and-step behavior of biped robots requires quickly extracting planar regions and obstacles with limited computing resources. To this end, this paper proposes an efficient method representing the environment as a hybrid of feasible planar regions and a heightmap. The feasible planar regions are used for footstep planning, preventing the body from hitting obstacles, and the heightmap is used to calculate foot trajectory to avoid foot collision during the swing process. The planar regions are efficiently extracted by leveraging the organized structure of points for nearest neighbor searches. To ensure safe locomotion, these extracted planar regions exclude areas that could cause the robot’s body to collide with the environment. The proposed method completes this perception process in 0.16 s per frame using only a central processing unit, making it suitable for look-and-step behavior of biped robots. Experiments conducted in typical artificial scenarios with BHR-7P and BHR-8P demonstrate its efficiency and safety, validating its effectiveness for the look-and-step behavior of biped robots.

## Introduction

Nature continuously inspires the development of biomimetic robots [1,2], with the biped robot serving as a prime example, modeled after the human structure, especially its locomotion [3]. These robots have substantial potential due to their unique ability to navigate uneven terrain [4] and perform complex manipulation tasks like humans [5], making them especially valuable in search and rescue missions within hazardous environments [6].

The look-and-step behavior is a strategy for biped robots that involves rapidly perceiving the surrounding environment, taking a step, and repeating the process without pause or deliberation [7]. This approach significantly enhances the robot’s adaptability to unknown environments and helps correct accumulated walking deviations during navigation.

During bipedal walking, biped robots must extract surrounding information about landing areas and obstacles. Landing areas are typically represented as planar regions, which are used for traversability checks for candidate footsteps, while obstacles provide critical data for collision avoidance and safe footstep planning in complex environments. Additionally, the processing time of the perception method is crucial, as the look-and-step behavior requires the robot to perceive the environment and replan footsteps within each swing cycle [7,8]. However, current methods for biped robots often need long computation times [9–11] or rely on additional computing units [8,12,13], such as graphics processing units (GPUs), to reduce processing time. Although GPUs enhance computational speed, their use adds weight and increases space requirements, making them less suitable for biped robots.

**Fig. 1.**
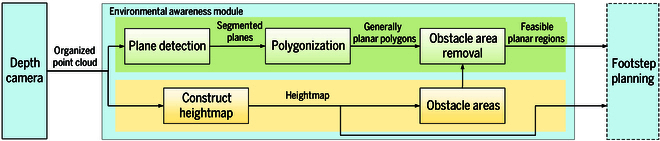
Outline of the proposed perception method.

**Fig. 2.**
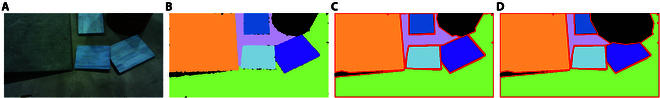
Feasible planar region extraction process. (A) Example scene: inclined planes with obstacles. (B) Plane detection result. (C) Polygonization result. (D) Obstacle area removal result.

In this paper, we propose an efficient method for representing the surrounding environment as a hybrid of feasible planar regions and a heightmap. The method consists of 2 subsystems: feasible planar region extraction and heightmap construction, as shown in Fig. 1. In the feasible planar region extraction subsystem, plane detection, polygonization, and obstacle area removal are performed sequentially, as illustrated in Fig. 2. Plane detection, which is the core of this method, efficiently segments planes using limited computing resources by leveraging the organization of point clouds (Fig. 2B). Based on the segmentation results, the contours of each segment are fitted into polygons (Fig. 2C). Obstacle areas are then removed from these polygons, identifying the feasible planar regions (Fig. 2D). The feasible planar regions, which are essential for safe footstep planning, and the heightmap, used to calculate the swing foot’s trajectory, together contribute to safe locomotion.

Combined with the footstep planning algorithm developed by our group [14,15], we experimentally verify the efficiency and safety of the proposed method in typical artificial scenes.

The contributions of this paper are as follows:

1. We propose an efficient method to present the environment as feasible planar regions and a heightmap within limited computing resources.

2. We apply the structured nature of points within an organized point cloud to the nearest neighbor search, which accelerates the planar extraction process.

3. We experimentally demonstrate that the proposed perception method can rapidly extract necessary environmental information while ensuring the robot’s walking safety.

The remainder of this paper is organized as follows. The next section discusses the related works about the perception method for bipedal walking. The “Perception Method for Look-and-Step Behavior of Bipedal Walking” section introduces the proposed method, and the “Plane Detection” section presents the process of plane detection. The “Experiments” section experimentally evaluates the proposed method and compares it with current approaches. In the “Discussion and Future Work” section, we discuss the shortcomings and future works of the proposed method. Finally, the “Conclusion” section concludes this paper.

### Related Work

Grid maps have been used in several studies to represent the environment. For example, some works [16–18] employed heightmaps, while Yamamoto and Sugihara [19] proposed a stochastic elevation-and-normal grid (ENG) map, where each cell encodes elevation, normal direction, and the standard deviation of measurements, enabling stable traversal of uneven terrain after traversability checks. Ueda et al. [20] adopted a fast plane detection algorithm [21] to extract planar information from depth images, representing its surroundings as 2-dimensional (2D) planar occupancy grids in 3D space. Jenelten et al. [22] used a heightmap to formulate a trajectory optimization problem that optimizes the base pose and footsteps simultaneously. Mastalli et al. [23] further optimized footstep locations, body motion, step duration, and footstep selection to enable navigation through complex terrains. However, grid maps are not well suited for footstep planning in continuous optimization, as they constrain the exploration of potential footstep plans compared to strategies that do not rely on predefined actions [24].

Several studies [25–27] have represented the environment as planar regions. However, the time required to extract these planar regions is often fairly significant. Bertrand et al. [9] presented the environment as an OctoMap [28] collected by the UTM-30LX-EW device and combined OctoMap with a nearest neighbor search approach to detect planar regions. This work needs several seconds (at least 6 s) to collect enough LIDAR scans for constructing an OctoMap. Thomas [10,11] represents the environment as a heightmap and extracts convex planes [29], a process that takes 2.5 s when working with point clouds containing far fewer points than typical depth images. Li et al. [30] construct informative planner regions for continuous bipedal walking. Lee and colleagues [31,32] developed a camera–laser fusion system to obtain a stable and dense point cloud for segmenting a specific object in an outdoor environment. The perceptual algorithm fitted the square planar regions from the environment by combining super-pixel segmentation with random sampling. This work requires 1.21 s on average and does not represent the actual contours of planar regions in the environment.

Other methods use GPUs to accelerate the process of extracting planar regions. For example, Fallon et al. [8] used the Kintinuous [33] algorithm to continuously fuse stereo imagery and applied a feature clustering-based method for terrain generation in footstep planning [24]. With acceleration from an NVIDIA GeForce GTX 680 GPU, the Kintinuous stereo fusion requires 0.11 s, and extracting the planar convex regions from the environment takes 0.615 s. The Florida Institute for Human and Machine Cognition (IHMC) [12] developed an algorithm that extracts planar regions from depth images using region growing and patch graphs. After GPU acceleration with an NVIDIA GTX 970, the algorithm processes the data in just 5 to 6 ms, enabling the biped robot to walk stably and continuously over irregular terrain [7] in combination with other related works [34–36]. Mishra et al. [13] construct planar surfaces from planar regions in each frame, using kinematic–inertial state estimates and accelerating the process with an NVIDIA GTX 1050Ti GPU. Bin et al. [37] propose a real-time planar semantic mapping algorithm optimized for NVIDIA RTX 4060 GPU acceleration, enabling efficient plane extraction and globally consistent map generation at over 30 Hz.

Kumagai et al. [38] represented the environmental information as a HeightField and an OctoMap, where the former is used for planar region detection and the latter for frequent collision checks. The footstep planner will re-plan footsteps using the current location during continuous walking. However, the average update duration of the HeightField is 2 s, which is too time-consuming to meet the requirements of continuous robot walking.

Compared to the works reviewed above, our proposed method can achieve fast environment representation using only a central processing unit (CPU), with a processing time of 0.16 s per frame. This is significantly smaller than the difference in time consumption between the robot’s swing cycle and footstep planning. The proposed method represents the environment as a hybrid of feasible planar regions and a heightmap, meeting the requirements for safe robot locomotion.

### Perception Method for Look-and-Step Behavior of Bipedal Walking

#### Efficient perception for look-and-step behavior of bipedal walking under limited computational resources

The environmental perception of bipedal walking focuses primarily on 2 essential aspects: landing areas and obstacles. Landing areas are critical for footstep planning, as they provide stable and feasible support for the robot’s feet [39,40]. Obstacles, on the other hand, are indispensable for collision avoidance during planning. Additionally, it is crucial to compute a collision-free swing trajectory for the swing foot based on the terrain, ensuring safe and effective movement in uneven environments.

The look-and-step behavior introduces further challenges, requiring re-extracting environmental information and re-planning footsteps in each swing cycle [7,8]. This imposes stringent demands on the computational efficiency of perception methods.

Existing methods for detecting landing areas often rely on planar region extraction techniques, which are computationally intensive. These methods either require excessive processing time [9,10] or depend on additional computational hardware to accelerate the process [12,13,37]. However, such hardware is unfriendly for robots due to limitations in internal space, load-bearing capacity, and cost.

This work extracts feasible planar regions and represents the surroundings as a heightmap. The extraction process is highly efficient as the ordered structure of points within an organized point cloud is utilized for nearest neighbor search. Feasible planar regions are utilized for footstep planning, with collision-prone areas excluded to ensure safe locomotion. The heightmap is then used to calculate the trajectory of the swing foot, preventing collisions with the environment.

#### Feasible planar region extraction

Feasible planar regions, with areas that could cause collisions between the robot and the environment already removed, ensure that the footsteps planned on these regions are collision-free. The detailed steps are as follows:

##### Plane detection

**Fig. 3.**
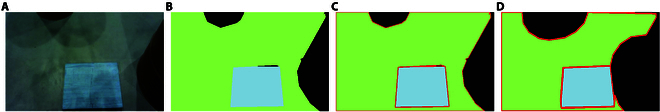
Feasible planar region generation process. (A) Real-world scene. (B) Plan detection result. (C) Polygonization result. (D) Result after removing dangerous areas.

Plane detection is a crucial step in generating feasible planar regions, enabling the identification of planar areas within organized point clouds. To improve efficiency, the proposed method leverages the structured nature of points within an organized point cloud to initialize a neighbor search graph. The detection result as shown in Fig. 3B. The details about this process are presented in the “Plane Detection” section.

##### Polygonization

After plane detection, the contours of the planar regions in 3D space need to be fitted. Due to the orderly arrangement of the detected planar points, their contours can be computed efficiently using OpenCV [41,42]. Ideally, these contours would directly represent the actual plane contours in the 3D scene. However, some contour point coordinates may be invalid due to the instability of depth measurements from a TOF (time of flight) camera. To address this, we employ the following equations to obtain reliable contour coordinates.

$$Z=\frac{-d}{\frac{a\left(u-c_{x}\right)}{f_{x}}+\frac{b\left(v-c_{y}\right)}{f_{y}}+c}$$ (1)

$$X=\frac{\left(u-c_{x}\right)Z}{f_{x}}$$ (2)

$$Y=\frac{\left(v-c_{y}\right)Z}{f_{y}}$$ (3)

where ${\left(X,\;Y,\;Z\right)}^{T}$ are the 3D coordinates of a contour point; $f_{x}$, $f_{y}$, $c_{x}$, and $c_{y}$ represent the camera intrinsics; *u* and *v* denote the pixel coordinates of contour point; and *a*, *b*, *c*, and *d* are the plane parameters. Specifically, ${\left(a,\;b,\;c\right)}^{T}$ is the normal of the plane, and *d* represents the perpendicular distance from the plane to the origin. The polygonization result is shown in Fig. 3C.

##### Obstacle area removal

**Fig. 4.**
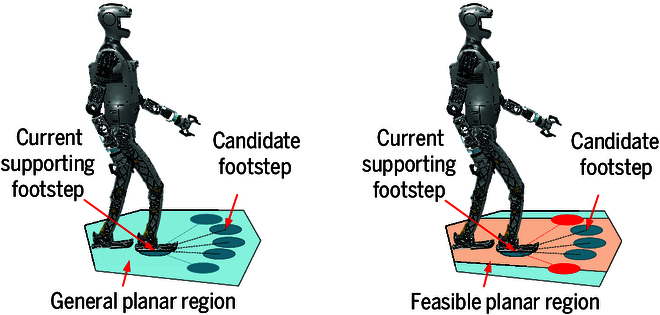
Node expansion is performed in general planar regions and feasible planar regions. Candidate footsteps (depicted as red circular areas) are quickly excluded if they lie outside the feasible planar region, which is generated by removing dangerous areas from the general planar region.

Collision checks are an integral part of the footstep planning process [19]. In continuous optimization-based footstep planning, obstacles are incorporated as constraints to ensure the safety of planned footsteps, with obstacle avoidance integrated into the optimization process using the contours of planar regions as input. In contrast, for discrete search-based footstep planning, collision checks are performed for each candidate footstep generated during node expansion, and those that result in collisions with the environment are excluded. To avoid frequent collision checks, we remove obstacle areas from the extracted planar regions, thus directly excluding candidate footsteps that may collide with the environment, as shown in Fig. 4.

To prevent body collisions during walking, the proposed method is inspired by the approach of setting an inflation radius on a 2D grid map for wheeled robot navigation. It removes areas that could cause the robot’s body to collide with obstacles when stepping on them. The inflation radius is determined based on the robot’s parameters. The result after removal is illustrated in Fig. 3D.

#### Heightmap construction

**Fig. 5.**
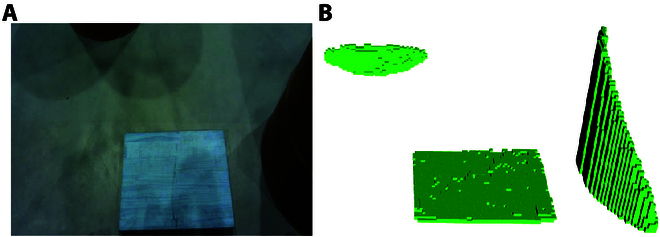
Construct heightmap. (A) Color image of the actual scene. (B) Corresponding heightmap.

**Fig. 6.**
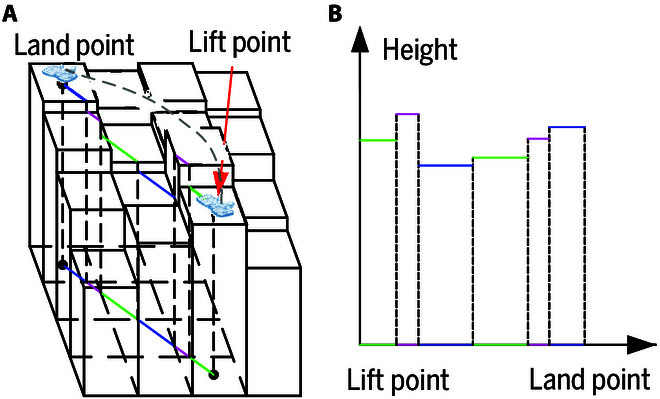
(A) Heightmap. (B) Staircase height curve of the barriers during swing process.

**Fig. 7.**
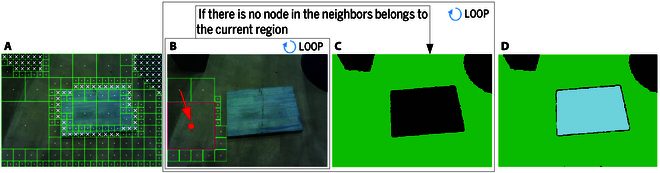
Plane detection. (A) Quadtree construction. (B) Region growing: The seed node, containing the most points, is identified and merged with its neighboring node that has the most points. The red box represents the seed node, and the green boxes represent its neighbors. The merging process continues until no neighboring nodes can be merged. (C) Result of region growing. (D) Result of plane detection: The final outcome of the plane detection process.

The heightmap is a 2.5D grid map, where each cell records the maximum height value of the points located within the corresponding cell. Constructed from the organized point cloud with a resolution of 0.01 m (Fig. 5), the heightmap is used to calculate a safe swing trajectory for the foot. The cells intersected by the line connecting the lift point and landing point are extracted from the heightmap, as shown in Fig. 6. Fig. 7 shows the staircase height curve, which represents the obstacles during the swing phase, calculated from the height values of these cells. This curve is useful for calculating an obstacle-free swing trajectory for the foot.

## Materials and Methods

### Plane Detection

Plane detection refers to the extraction of planar regions from organized point clouds. The detailed process is illustrated in Fig. 7.

#### Quadtree construction

The quadtree can efficiently store organized point cloud in a 2D space. In this process, a quadtree is used to present the organized point cloud and the planar information of its leaf nodes is calculated, avoiding the need for per-point normal estimation. Each leaf node corresponds to a square region in the organized point cloud and allows for fast retrieval of points within this region using their row and column indices along with the leaf node’s width. The proposed method uses a bottom-up approach to determine whether an internal node is a planar node, where the planar information of child nodes is utilized for this decision. When an internal node is identified as a planar node, its child nodes are removed.

Specifically, first, the depth of the quadtree d and the width of the quadtree $W_{T}$ are calculated according to the width of the leaf node $W_{l}$ and the size of the organized point cloud $W_{I}\cdot H_{I}$ based on

$$\begin{array}{c} d=\left\lceil {\text{log}}_{2}^{\text{max}\left(W_{I},\;H_{I}\right)/W_{l}}\right\rceil \end{array}$$ (4)

$$W_{T}=W_{l}\cdot 2^{d}$$ (5)

where $\left\lceil \cdot \right\rceil$ represents rounding up.

The organized point cloud is divided into nodes recursively until the depth of the quadtree is reached.

Principal components analysis (PCA) is applied to a leaf node to determine whether it is a planar node and to compute its planar information. Following the approach in Ref. [43], a more efficient routine [44] is used to decompose the eigenvalues of a 3 × 3 symmetric matrix. The scatter matrix $S_{3\times 3}$ of a leaf node is computed as:

$$S_{3\times 3}=\sum_{i=1}^{k}\left(p_{i}-c\right){\left(p_{i}-c\right)}^{T}$$ (6)

where c is the center of the node, $p_{i}$ is a point in the node, and *k* is the number of points in the node. The proposed method uses PCA to calculate the eigenvalue $\lambda_{1}$, $\lambda_{2}$, $\lambda_{3}$ $\left(\lambda_{1}\leq \lambda_{2}\leq \lambda_{3}\right)$ of matrix $S_{3\times 3}$. A leaf node is considered a planar node if the following inequality is satisfied

$$\frac{\lambda_{1}}{\lambda_{1}+\lambda_{2}+\lambda_{3}}\leq T_{e}$$ (7)

where $T_{e}$ is the threshold for determining whether a leaf node is planar, based on the noise in the organized point cloud and the width of the leaf node. The normal of the node is the eigenvector corresponding to the smallest eigenvalue.


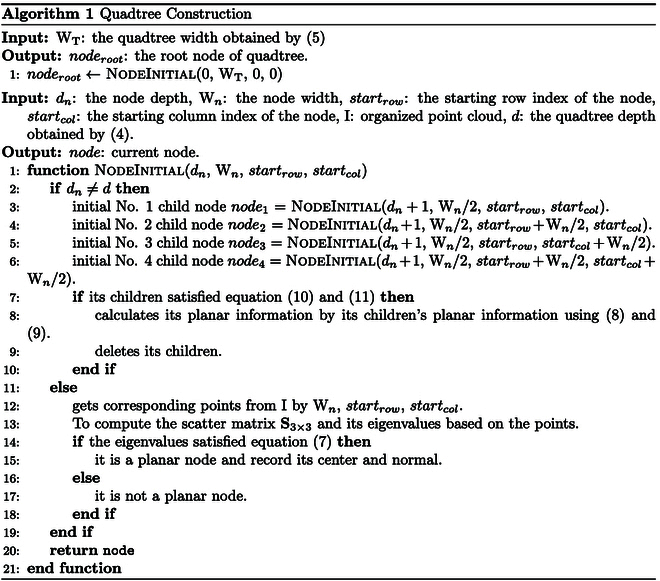


However, for an internal node, the planar information of its children is used to determine whether it is a planar node. The mean normal $n_{m}$ and the center $c_{m}$ of a parent node are first computed by

$$n_{m}=\left(\sum_{i=1}^{4}w_{i}\cdot n_{i}\right)/\sum_{i=1}^{4}w_{i}$$ (8)

$$c_{m}=\left(\sum_{i=1}^{4}w_{i}\cdot c_{i}\right)/\sum_{i=1}^{4}w_{i}$$ (9)

where $n_{i}$ and $c_{i}$ are the normal and center of child *i*, and $w_{i}$ is the weight of child *i*, represented by the number of points in the child node. The children are considered to belong to the same plane if the following inequality constraints are satisfied:

$$n_{i}\cdot n_{m}\geq T_{nm}$$ (10)

$$\left|n_{m}\cdot \left(c_{m}-c_{i}\right)\right|\leq T_{dm}$$ (11)

where $T_{nm}$ is the tolerance for angular similarity between the mean normal and the normal of each child node, and $T_{dm}$ is the threshold for the maximum orthogonal distance from a child node to its parent node. These thresholds are determined like the way of $T_{e}$ in Eq. (7).

If the above constraints are met, the parent node is a planar node and its planar information are directly calculated from its children’s planar information using Eqs. (8) and (9), rather than from the points in the internal node itself, which improves computational efficiency.

The details of quadtree construction are outlined in Algorithm 1.

#### Node graph construction

**Fig. 8.**
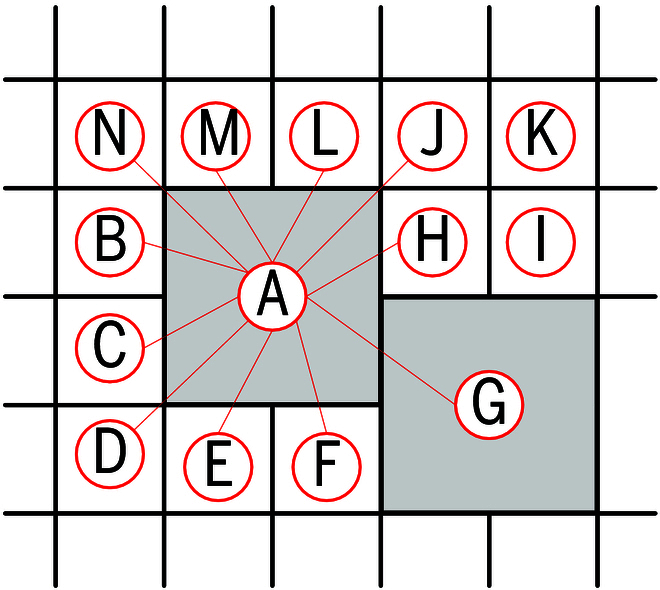
Node graph construction. The nodes can be arranged based on their row and column indices and width, which allows for determining the adjacency relationships between the nodes. For example, the nodes adjacent to node A are B, C, D, E, F, G, H, J, L, M, and N.

Based on the description above, all nodes within the quadtree are identified. The adjacency of nodes is determined by their arrangement in the organized point cloud, similar to the way pixels are arranged in an image. As shown in Fig. 8, if 2 nodes are adjacent, an edge is established between them.

#### Region growing

Region growing is performed based on the constructed node graph, following these steps: (a) select a planar node containing the most points from the node graph as the seed node for the current region, (b) merge the neighboring planar node with the most points (if no planar nodes can be merged, we proceed by merging points from nonplanar nodes; this process repeats until no nodes or points remain to be merged; additionally, the planar information of the current region is updated whenever a node or point is added). and (c) remove all points already clustered from the node graph. The detailed region growing process is presented in Algorithm 2. Both merging planar nodes and adding points must meet the following conditions.


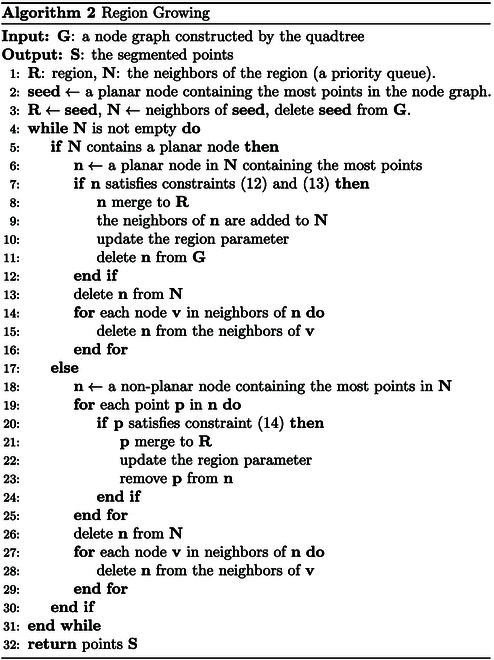


When considering whether a planar node can be merged, the following inequality constraints must be satisfied:

$$n_{n}\cdot n_{r}\geq T_{nr}$$ (12)

$$\left|n_{r}\cdot \left(c_{r}-c_{n}\right)\right|\leq T_{dr}$$ (13)

where $n_{n}$ and $c_{n}$ are the normal and the center of the planar node, and $n_{r}$ and $c_{r}$ are the normal and the center of the current region. The meanings and determination methods of $T_{nr}$ and $T_{dr}$ are similar to those of $T_{nm}$ and $T_{dm}$ in Eqs. (10) and (11).

For a nonplanar node, whether a point within the node belongs to the current region is determined by satisfying the following inequality constraint

$$\left|n_{r}\cdot \left(c_{r}-p\right)\right|\leq T_{dp}$$ (14)

where p is a point in the nonplanar node and the threshold $T_{dp}$ represents the maximum allowable distance from the point to the current region. This threshold is determined based on the noise of the organized point cloud.

### Experiments

#### Platform

**Fig. 9.**
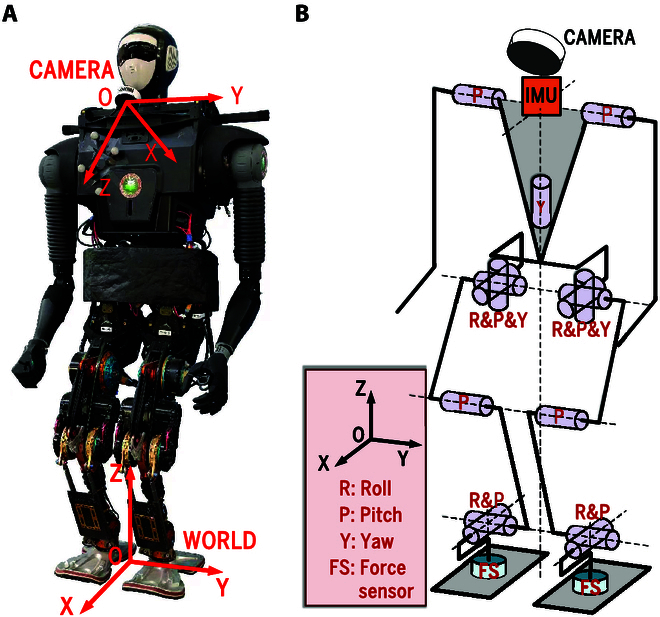
Depiction of BHR-7P. (A) BHR-7P. (B) Abstracted model of BHR-7P.

**Fig. 10.**
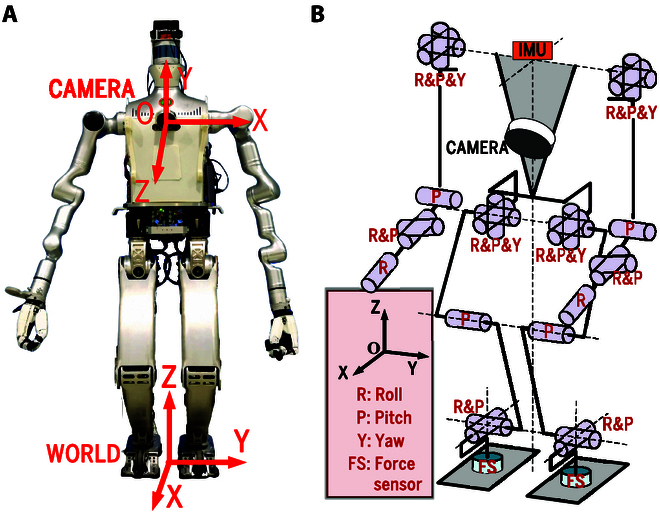
Depiction of BHR-8P. (A) BHR-8P. (B) Abstracted model of BHR-8P.

BHR-7P is a position-controlled biped robot that weighs 55.9 kg and stands 1.4 m tall. It has 15 degrees of freedom (DoFs) for the whole body, which are 6 DoFs per leg, 1 DoF for each shoulder, and 1 DoF for the waist (Fig. 9). A PC104 computer with a 1.99 GHz Intel Celeron J1900 processor is used to control the robot, and the control rate is 200 Hz. BHR-8P is a position-controlled biped robot, which weighs 72 kg and stands 1.6m tall. It has 26 DoFs for the whole body, which are 6 DoFs per leg and 7 DoFs per arm (Fig. 10). The control computer is a NUC8 with a 2.3 GHz Intel i5-8259U processor, and the control rate is 250 Hz. The installed camera is an Intel RealSense L515 [45], which generates 640 × 480 organized point clouds with high accuracy at 30 Hz. The camera [with a FOV (field of view) of $75^{\circ }\times$ $55^{\circ }$] is installed at 1.25 m height. The camera on BHR-7P is mounted at an angle of $27.5^{\circ }$ relative to the vertical direction, while the installation angle on BHR-8P is $45^{\circ }$. This camera’s short exposure time of less than 100 ns per depth point enables it to capture rapidly moving objects with minimal motion blur, making it suitable for dynamic data collection scenarios.

The proposed perception method is implemented on a mini-personal computer UM790Pro using an AMD 7940HS of 4.0 GHz and 16 GB RAM. In its implementation, no parallelism was employed, whether OpenMP, GPU, or multi-threading.

#### Critical parameters

Several parameters play a critical role in determining the time consumption of the proposed method and the accuracy of the contours of feasible planar regions. Among them, the most influential parameter is the width of the leaf node. If this value is too large, the detection time decreases to some extent; however, the jaggedness of the planar pixel regions becomes more pronounced, reducing the accuracy of the general planar regions’ contours. Conversely, if the value is too small, the jaggedness of the planar pixel regions is suppressed, but the calculation time increases. Our experiments indicate that a leaf node width of 5 pixels provides an optimal balance between the accuracy of general planar regions’ contours and the computational efficiency of our method.

To mitigate the impact of jaggedness on the accuracy of the planar pixel regions’ contours, we employ the approxPolyDP function in OpenCV [41,42] to compute approximate contours. The distance from the approximate contour to the actual contour is set to 5 pixels, as experiments have shown that this value effectively balances contour accuracy and computational efficiency.

Additional thresholds discussed in the paper are detailed in Table 1. Parameters $T_{e}$, $T_{dm}$, $T_{dr}$, and $T_{dp}$ are associated with point cloud noise, which is influenced by the distance of points from the origin. The variable *d* in Table 1 represents the distance from a point or node to the origin. In contrast, thresholds $T_{nm}$ and $T_{nr}$ are typically determined based on the specific scene and experimental experience. These thresholds are generally slightly smaller than the smallest angle between 2 intersecting planes in the scene. In different scenarios, minor adjustments to these thresholds are made as needed.

**Table 1.** Values of thresholds

Threshold	Which equation used	Value
$T_{e}$	(4)	$0.001\times d+\left(0.008∼0.012\right)$
$T_{nm}$	(7)	0.95∼0.985
$T_{dm}$	(8)	$0.001\times d+\left(0∼0.004\right)$
$T_{nr}$	(9)	0.95∼0.985
$T_{dr}$	(10)	$0.001\times d+\left(0∼0.004\right)$
$T_{\mathrm{dp}}$	(11)	0.001×d+0.003

#### Experiment on real-world environment

To verify the effectiveness of the proposed method, we test it in 2 typical scenes combined with our stability controller [46–48]. The footstep planning algorithm [15] used in the following experiments is based on greedy and heuristic optimization. It considers kinematic constraints resulting from robot parameters. The algorithm plans the most suitable footsteps for our robot to reach the goal within the feasible planar regions. Since heightmap is a universal map expression structure, it is not illustrated for each experiment.

##### BHR-7P robot climbing stairs

**Fig. 11.**
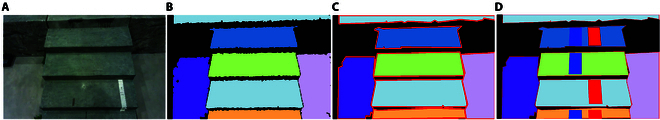
(A) Example scene: stairs. (B) Plane detection result. (C) Polygonization result. (D) Results of footstep planning.

**Fig. 12.**
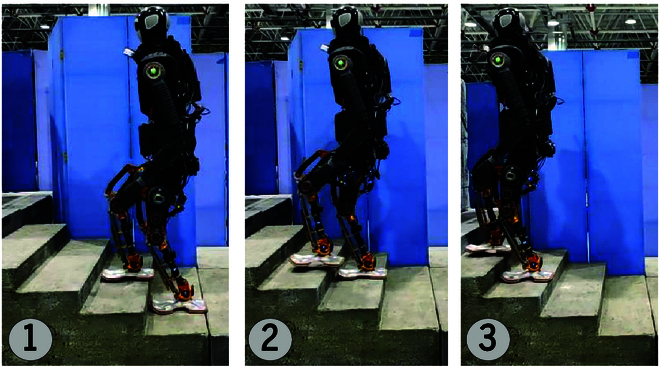
BHR-7P climb-up stair experiment.

Stairs are very common in an artificial environment. In this scenario, the width of the stairs is 1 m, the tread depth is 0.27 m, and the riser is 0.1 m. The planar pixel detection results (Fig. 11B) reveal that in some shadow areas of the stairs, the quality of the organized point cloud is poor, resulting in jagged edges of the segmented planes. According to the detection results illustrated in Fig. 11, BHR-7P can climb the stairs independently. This experiment verifies that the planar pixel detection algorithm and polygonization can meet the requirements of discontinuous terrain without obstacles (Fig. 12).

##### BHR-8P traversing steps and a slope with obstacle

**Fig. 13.**
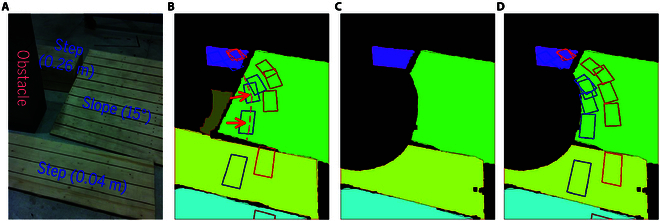
(A) Example scene: steps and a slope with an obstacle. (B) Plan detection result and the planned footsteps before removing dangerous areas. The red arrows indicate several unsafe footsteps that could potentially cause collisions between the robot’s body and the environment. (C) Feasible planar regions after removing dangerous areas. (D) Planned footsteps within feasible planar regions.

**Fig. 14.**
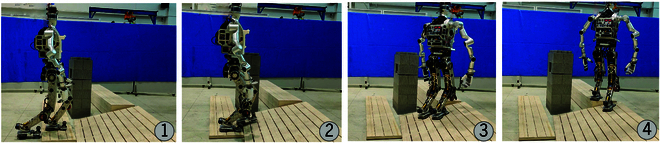
Traversing through steps and a slope with an obstacle.

In this scenario, there are 2 steps, 1 slope, and 1 obstacle. The step heights are 0.04 and 0.26 m, respectively, and the slope has an angle of 15${}^{\circ }$, as shown in Fig. 13A. Fig. 13B presents the plane detection results, where planes with excessive angles relative to the ground have been discarded. Although both the obstacle and the distant ground are planar, the quality of the point cloud is degraded due to the absorptive properties of the obstacle and the distance to the camera, which results in the failure of plane detection in these regions. Fig. 13C shows the traversable area after removing these hazardous regions, where stepping would cause a collision with the environment. Fig. 13D illustrates the planned footsteps within the traversable areas, ensuring the robot can safely reach the goal by following the planned footsteps. In contrast, footsteps planned without removing hazardous areas may place landing points within these dangerous regions. This can lead to collisions between the robot’s body and the environment, which is unsafe for the robot’s locomotion, as shown in Fig. 13B. Fig. 14 demonstrates the robot navigating this scenario.

#### Comparison of time consumption with other works

We compared the time consumption of the proposed method with other works. The corresponding results are reported in Table 2. The perception methods that express the environment as a grid map are not taken into consider since the grid map is unsuitable for footstep planning of continuous optimizations. The proposed method simultaneously represents the surrounding environment as a hybrid form of feasible planar regions and a heightmap, making it highly suitable for robot walking. Unlike the planar regions detected by competing perception methods, the feasible planar regions generated by our approach help prevent collisions between the body and the environment, thereby improving overall navigation safety. As shown in Table 2, Lee and colleagues [31,32] do not represent the actual contours of the planar regions in the environment. For time consumption, Bertrand et al. [9] need excessive time consumption. Although the works [8,12,37] require relatively short time consumption, they use extra computing units to reduce it and ignore the environmental obstacle. The time consumption of the proposed method is 0.16 s at most only using a CPU, which is less than the difference between the swing cycle (1.2 s) and the footstep planning cycle (0.3 s).

**Table 2.** Comparison of the proposed method against existing perception methods

Related work	Input format	Time/s	Obstacle information	Computing unit
Fallon et al. [8]	3D grid	0.615	×	3.30 GHz with GTX 680
Mishra et al. [12]	Organized point cloud	0.005	×	3.50 GHz with GTX 970
Bin et al. [37]	Organized point cloud	<0.02	×	5.40 GHz with GTX 4060
Bertrand et al. [9]	OctoMap	>6	×	Unknown
Lee et al. [31,32]	Point cloud	1.21	×	3.60 GHz
Our method	Organized point cloud	0.16		4.0 GHz

## Discussion

While the proposed method demonstrates effectiveness in planning footsteps for biped robots on planar regions, there are several limitations that should be considered. First, the method depends on empirically set thresholds tailored for specific scenarios. These thresholds, while effective in the tested scenarios, may not automatically adapt to different environments or terrain variations. However, this is a manageable challenge that could be addressed through future work on adaptive thresholding techniques.

Additionally, the current implementation plans multiple footsteps based on the proposed perception method results and executes them continuously without perception during execution. This perception method inherently supports look-and-step behavior by achieving relatively short processing times under limited computational resources. This capability serves as a crucial foundation for enabling look-and-step behavior in more dynamic and complex environments. Future work will focus on extending this approach to continuous walking based on the look-and-step paradigm, further enhancing the robot’s adaptability to changing scenarios.

Furthermore, the environment perception in this study was conducted using L515, which has a relatively narrow field of view and produces low-quality point clouds under certain conditions. These limitations could affect the robot’s ability to accurately perceive and adapt to diverse terrains. To further enhance the robot’s adaptability and robustness in varied environments, future work could explore the use of industrial cameras with a wider field of view and more stable point cloud outputs.

## Conclusion

In this work, we propose an efficient and safe perception method tailored for the look-and-step behavior of bipedal robots. The method represents the environment as a hybrid model of feasible planar regions and a heightmap. The planar regions, used for footstep planning, are designed to be safe, as areas likely to cause collisions with the environment are excluded. The heightmap is used to plan the trajectory of the foot, allowing the robot to avoid obstacles during the swing phase. The proposed method rapidly extracts planar regions using the organization of depth image pixels to optimize the nearest neighbor search in the planar region detection process. The proposed method processes each frame in 0.16 s using only a CPU, making it practical for biped robots. Tests in typical artificial scenarios with BHR-7P and BHR-8P demonstrate its efficiency and safety for the look-and-step behavior of biped robots.

## Data Availability

The data supporting the findings of this study are available at https://github.com/lichao951787328/paper_source_data.git.
